# Editorial: Deceiving the host: mechanisms of immune evasion and survival by pneumococcal bacteria

**DOI:** 10.3389/fcimb.2023.1231253

**Published:** 2023-06-19

**Authors:** Karthik Subramanian, Anirban Banerjee

**Affiliations:** ^1^ Pathogen Biology Division, Rajiv Gandhi Centre for Biotechnology, Thiruvananthapuram, Kerala, India; ^2^ Indian Institute of Technology Bombay, Mumbai, India

**Keywords:** immune evasion, host-pathogen interaction, pneumococcal vaccine, complement, glycosidases, pneumococcus (*Streptococcus pneumoniae*)

*Streptococcus pneumoniae* (the pneumococcus) colonizes the human upper respiratory tract and is the leading cause of respiratory tract infections around the world killing over a million people worldwide (Collaborators, 2018). Globally, *S. pneumoniae* is the major causative of infection-related deaths in children below 5 years (McAllister et al., 2019) (Collaborators, 2022), and the elderly above 70 years. Pneumococcus colonizes the nasopharynx of 27–65% of children and 10% of adults asymptomatically (Yahiaoui et al., 2016). However, bacterial spread to the lower respiratory tract and other organs results in pneumonia, septicemia, meningitis, and otitis media (Cherazard et al., 2017) collectively referred to as invasive pneumococcal diseases (IPD). Treatment of pneumococcal infections is becoming complicated due to fast emerging resistance to currently used antibiotics such as beta-lactams, macrolides, and tetracyclines. Antibiotic misuse and horizontal transfer of antibiotic-resistance genes have resulted in the alarming growth and spread of antibiotic resistance amongst pneumococcal strains. In 2017, WHO declared pneumococcus as one of the top 12 global priority pathogens that urgently requires research into new antibiotics. Current vaccines have reduced incidence of IPD caused by vaccine serotypes, but emergence of non-vaccine serotypes is an emerging problem. With over 100 pneumococcal serotypes, broad-spectral or serotype independent vaccines are required for the prevention of pneumococcal diseases. Given these pressing issues, there is an urgent requirement to decipher pathogenesis and immune evasion mechanisms of pneumococcus for development of novel therapeutics. In this Research Topic, we have compiled a series of articles focusing on different aspects of pneumococcal pathogenesis and novel vaccine approaches that are summarized below.

Parveen and Subramanian analyze the roles of bacterial and host-derived vesicles in the pneumococcal diseases and their clinical applications as diagnostics and therapeutics. Bacterial vesicles derived from outer membrane contain antigenic virulence factors and are taken up by host cells resulting in pro-inflammatory signalling. Immunization of mice with bacterial vesicles induces neutralizing antibodies and confers protection against infection. Outer membrane vesicles released from genetically engineered *Neisseria meningitidis* expressing endotoxin and *E. coli* expressing pneumococcal capsule have been tested as potential vaccines. However, problems with scalability and batch to batch variation have remained as obstacles, precluding their application for further clinical assessment. Host-derived vesicles could be engineered with therapeutics and antibodies for targeted drug delivery. Exosomes engineered with integrin β4 and loaded with tumor suppressing miRNA inhibited lung cancer proliferation and metastasis *in vivo*. EVs isolated from patient biofluids such blood, lung fluid and saliva could be used to develop vesicle-based diagnostic kits that discriminate viral and bacterial pneumonia due to their unique protein content.

Gil et al. discuss the interactions between *S. pneumoniae* and the complement system and the mechanisms by which these bacteria evade complement killing. Classical component of the complement pathway is activated through direct C1q binding, antibody recognition of cell wall, binding of serum proteins such as C-reactive protein and serum amyloid protein P and interaction of capsule with macrophage receptors. The role of the lectin pathway in pneumococcal clearance is ambiguous with contradictory data from knockout mice showing limited role of some lectins such as mannose binding lectin, but importance of certain others such as mannan-binding lectin associated serine protease-2. Pneumococci have evolved several mechanisms to evade complement killing such as the polysaccharide capsule, prevalence of diplococcal form as opposed to chains, complement consumption by pneumolysin, and choline binding proteins such as PspA and PspC that inhibit complement. Non-capsular dependent variations can also occur between serotypes owing to allelic variations in choline binding proteins. Further studies are required to understanding roles of different virulence proteins on complement evasion in IPD.

Mathew et al. explored the role of extracellular glycosidases in pneumococcal immune evasion. Glycosidases cleave terminal or internal glycan residues from host glycoproteins and receptors thereby rendering proteins inactive or support bacterial growth by utilizing released carbohydrates. Pneumococci express different types of glycosidases such as neuraminidases, β-galactosidases and N-acetylglucosaminidases. While the neuraminidases, NanA, NanB and NanC cleave α2-3 or α2-6 linked terminal sialic acid residues, the β-galactosidases, BgaA and BgaC cleave galactose linked to N-acetylglucosamine or glucose through β1-4 or β1-3 linkages. NanA, BgaA, StrH and EndoD can sequentially hydrolyze N-linked glycans on host proteins to support bacterial growth in chemically defined medium lacking glucose. Pneumococcal glycosidases also contribute to colonization and biofilm formation by promoting bacterial adhesion to host receptors, facilitating nutrient acquisition and mediating bacterial aggregation. Deglycosylation of host defense proteins such as complement proteins, lactoferrin and cleavage of mucosal IgA by IgA protease enables pneumococci to evade immune responses. Thus, glycosidases promote pneumococcal virulence by versatile mechanisms during different stages of bacterial pathogenesis and are therefore attractive therapeutic targets.

Silva et al. review the status of novel vaccine platforms for *S. pneumoniae* which are not based on the capsular polysaccharide. Due to serotype-restricted protection offered by the current polysaccharide PPSV and PCV conjugate vaccines and consequent replacement by non-vaccine serotypes, there is an urgent need to develop non-capsular based vaccines. Protein-based vaccines comprised of the conserved antigens-PspA, PcpA, PhtD and inactivated whole-cell based vaccine based on D39 strain lacking capsule are currently in clinical trials. The inactivated whole cell vaccine SPWCV comprising multiple conserved antigens showed promising results in phase 1 trial by inducing both cellular and humoral responses. The chimeric vaccine, designated PSPF, composed of epitopes from PsaA, PspA, Spr1875 and FliC, induced protective antibodies and reduced bacterial burden. Another vaccine designated ASP3772 developed using a multiple antigen presenting system platform, and consisting of 24 biotinylated polysaccharides fused to two surface proteins, induced specific antibodies and protective Th17 responses. Finally, the successful use of COVID-19 RNA vaccine has opened up opportunities to develop an RNA-based pneumococcal vaccine consisting of several important antigens in the future.

Invasive pneumococcal diseases pose a great threat to the global health and economy that needs immediate attention amidst the recent surge in pandemics. The four articles published in this Research Topic offer new insights into different facets of pneumococcal infections and control such as molecular pathogenesis, immune evasion and novel vaccine strategies. We believe that these articles summarize the recent advancements in understanding pathogenesis mechanisms and open up new avenues for pneumococcal disease control that need to be addressed in the future studies. We sincerely thank all the authors and reviewers for their contributions to this Research Topic.

## Author contributions

All authors have made a significant, direct and intellectual contribution to the work and approved it for publication.
